# Identifying the Associated Risk Factors of Sleep Disturbance During the COVID-19 Lockdown in Bangladesh: A Web-Based Survey

**DOI:** 10.3389/fpsyt.2020.580268

**Published:** 2020-09-17

**Authors:** Tasnim Ara, Md. Mahabubur Rahman, Md. Abir Hossain, Amir Ahmed

**Affiliations:** ^1^ Institute of Statistical Research and Training, University of Dhaka, Dhaka, Bangladesh; ^2^ Department of Chemical and Food Engineering, Dhaka University of Engineering and Technology, Gazipur, Bangladesh; ^3^ Department of Nutrition and Food Engineering, Daffodil International University, Dhaka, Bangladesh

**Keywords:** COVID-19, lock down, home confinement, sleep disturbance, anxiety, AOR, AUC

## Abstract

**Background:**

Bangladesh, a developing country with a lower-middle-income and one of the world’s most densely populated areas, has been severely affected by COVID-19. This global epidemic is not only affecting the physical health of the patients but also causing severe psychological effects among those who have not yet been infected. Sleep disturbance is one of the key symptoms of major depression and one of the proven risk factors for suicide. The objective of this study is to identify the risk factors associated with sleep disturbance which has developed as a general impact of COVID-19 and new normal life during the lockdown (a measure to control the spread of COVID-19) in Bangladesh.

**Methods:**

Demographic characteristics, COVID-19, and lockdown related information have been collected from 1,128 individuals by conducting a web-based survey. Respondent’s perspective regarding sleep disturbance during COVID-19 lockdown is considered as the outcome of interest which is dichotomous. Descriptive statistics methods have been applied to explore the distribution of respondent’s demographic characteristics. Pearson’s chi-square tests have been performed to relate the sleep disturbance status of the respondents to their demographic, personal, and COVID-19 related information. Furthermore, a multivariable logistic regression model has been adopted to identify the significant association of sleep disturbance with the demographic, COVID-19, and lockdown related information of respondents during the COVID-19 lockdown in Bangladesh.

**Findings:**

The prevalence of sleep disturbance during the COVID-19 lockdown is found to be higher among participants aged 31–40 years. Gender disparity has also been observed in favor of male participants, whereas no significant regional heterogeneity has been found. Working from home or doing online classes during the lockdown has been found as a potential predictive factor of sleep disturbance. Losing a job has been considered as an adverse economic effect of COVID-19, which also induces sleep disturbance. Perception regarding the risk of getting infected and anxiety triggered the chance of developing sleep disturbance. The sleeping schedule is also found as a risk factor for sleep disturbance.

**Conclusion:**

Evidence-based policies are required to combat psychological challenges that have arisen due to COVID-19, primarily targeting the groups who are largely suffering from sleep disturbance.

## Introduction

The emergence of a cluster of acute respiratory illnesses that occurred by exposure to SARS-COV-2 is officially identified as COVID-19, which was first observed in December 2019 in Wuhan, Hubei Province of China (1–3). The WHO declared the COVID-19 outbreak as a “pandemic” on March 11, 2020 as the virus spreads increasingly worldwide (4). As of June 24, 2020, 213 countries and territories around the world are affected by the SARS-COV-2, and a total of 9,360,758 COVID-19 cases in the world have been confirmed, and 479,896 deaths have occurred worldwide from the disease (5). Almost all countries are adopting preventive measures such as remote office activities, international travel bans, mandatory lockdowns, and physical distancing. Bangladesh, a developing country with a lower-middle-income and one of the world’s most densely populated areas, is also trying to stop the spread of the disease with its limited resources. The country confirmed the first COVID-19 case on March 7 (6). There are 119,198 total confirmed cases and 1,545 total deaths in Bangladesh as of June 24, 2020 (7). The government of Bangladesh declared the enforcement of lockdown on March 26 to prevent the spreading of this infectious virus (6).

There is a considerable relation between mental health and poverty (8). Low and middle-income countries have a higher burden of mental disorders than economically developed countries (9, 10). Mental health resources include policy and infrastructure within countries, mental health services, community resources, human resources, and funding. In low-and middle-income countries, mental health services are highly insufficient, and the available resources for mental health are still scarce, inequitably distributed, and inefficiently utilized (11, 12). Compared to Australia and Canada (two high capacity countries in terms of mental health response), Bangladesh is more than 100-fold behind in terms of the number of psychiatrists per 100,000 population (13). The rate of occupational therapists per 100,000 population is 0 in Bangladesh, whereas this rate is respectively 7.65 and 3.7 in Australia and Canada (13). In Australia and Canada, people with mental disorders pay at least 20% of the cost of mental health care, whereas in Bangladesh patients pay entirely out of their own pocket to receive the service (13). The only nationally representative survey conducted between 2003 and 2005 illustrated the high burden of mental disorders in Bangladesh (14). Notedly, mental health services are virtually non-existent at the primary care level throughout the country (15, 16). There is a considerable lack of an adequate number of psychiatrists, and they are mostly located in big cities which makes the burden heavier (17). In such a situation, mental health issues during the COVID-19 pandemic might be severe in Bangladesh.

The mass home confinement since the COVID-19 outbreak in December 2019 has developed a stressful situation for many across the globe. COVID-19 not only affects the physical health of the population but also has a serious impact on mental health (18, 19). In addition, symptoms of anxiety and depression and self-reported stress are common psychological effects of the COVID-19 pandemic (20). Previous studies have also shown that the prevalence of novel infectious diseases, such as severe acute respiratory syndrome (SARS) can increase anxiety, depression, and stress levels in the general population (21). Being forced to stay at home, work from home, do home-schooling for children, severely reduced social interaction, work many more hours in stressful situations, and health risks can have a severe impact on daily activities and nighttime sleep (19). Even if people under lockdown have less possibility to develop an infection they often suffer from negative psychological effects, which may disrupt the sleep quality (22).

Sleep plays a fundamental role in emotion regulation (23–26). Many cross-sectional epidemiological studies have indicated that sleep disturbance is closely associated with new-onset of poor mental health status and lasting poor mental health status (27–31). Disturbed sleep is also considered as a causal factor in the occurrence of many mental health disorders (32). Several studies have shown that proper quality sleep not only reduces the risk of non-communicable diseases (NCDs) (33–38) but also helps to improve immunity to viral infection (39, 40). Thus, through good quality sleep, a better immune system can be developed which in turn may have an impact on the susceptibility of COVID-19 infection. Psychological wellbeing and sleep are affected by several socio-economic factors such as economic burden, family support, and social support (41). Recently, several studies have investigated the influence of social factors and factors related to COVID-19 on sleep quality in China and European countries (18, 19, 42). This paper attempts to assess the risk factors associated with sleep disturbance in the context of COVID-19 and new normal life during the lockdown in Bangladesh.

## Materials and Methods

### Study Design and Participants

A web-based self-reported cross-sectional survey has been conducted to collect the data. This web-based survey was intended to reach as many individuals as possible. Thus, rather than using the official psychological tools to respond to questions about sleep disorder or anxiety, to keep the questionnaire easy and understandable to the respondents and to make the interview duration shorter, we constructed a self-reporting questionnaire. This is the most widely used tool in community surveys for physical and mental health evaluation (43–45). Features that might be related to sleep quality evaluated by previous studies were included in the questionnaire (46–48). Moreover, some hypothesized risk factors of sleep disturbance in the context of COVID-19 and new normal life during the lockdown were also included in the questionnaire. The survey questionnaire with the consent form was shared on the internet through different social networking and messaging site (Facebook, Whatsapp, Viber, IMO, *etc.*) and e-mail, SMS. All Bangladeshi people using these tools may see this survey and may answer the questionnaire by clicking the relevant link. This web-based questionnaire was completely voluntary and non-commercial.

### Data Collection

Participants answered the questionnaires anonymously on the Internet from May 12, 2020, to May 18, 2020. All subjects reported their demographic data, COVID-19 related information, and questions related to sleep quality. To ensure the quality of this survey, questions were provided in both Bengali and English language and encouraged participants to answer carefully through questionnaire explanations. A total of 1,150 individuals have participated in the survey and among them, 1,128 individuals have completed the questionnaire. Incomplete responses have been excluded from the analysis.

### Variables Assessed and Measured

#### Demographic and Other Personal Information

Demographic variables include administrative division, place of residence (“urban”, “rural”), religion, age, gender, marital status, educational level, employment status, and income level. In addition, family-size and body mass index (BMI) of the participants have also been included.

#### Information Regarding COVID-19 and New Normal Life During the Lockdown

Individuals were asked about different pieces of information regarding COVID-19 and their new normal life during the lockdown. This information includes whether they are following the social distancing rule; whether they or their family members, relatives, friends, or neighbors got infected by COVID-19; whether they are working from home/doing online classes; whether they have to go to the workplace during the lockdown; whether any of the family members including respondent have lost their job; exercise status, whether food consumption dominates the new normal life during the lockdown, daily internet usage, perception regarding the risk of getting infected by COVID-19, anxiety, sleeping schedule, *etc.*

#### Outcome Measurements

Sleep disturbance was considered as a binary outcome of interest. Individuals were asked whether they are facing any kind of problems or disturbances in their sleep during the lockdown period or not.

### Statistical Analysis

Descriptive statistical methods have been applied to assess the distribution of the demographic characteristics of the Bangladeshi population. Then, Pearson’s chi-square tests (49) were used to find associations between independent variables and sleep disturbance, with those variables showing an association of 0.05 selected to be the part of the model. A multivariable logistic regression (50, 51) has been carried out to find associations between the independent variables, the dependent variable, and adjust for confounders. The adjusted odds ratio (AOR) and 95% confidence interval (95% CI) have been obtained from the logistic regression model. In the logistic regression model, coefficients with *p*-values (2-sided tests) less than or equal to 0.05 have been considered as statistically significant (5% level of significance). As a measure of model performance, the area under the curve (AUC) of the receiving operating characteristic (ROC) has been calculated along with its standard error by using its equivalence to the Wilcoxon statistic (52, 53). All data were analyzed using R (Version 3.6.2, RStudio version: 1.1.383) and STATA version 14.0 (Stata SE 14, Stata Corp, College Station, TX, USA).

## Results

The socio-demographic information of the respondents is presented in **Table 1**. The analysis was based on a sample of 1,128 respondents (male 55.1% and female 44.9%). The majority of the respondents (75.71%) were from the second age group (21–30 years). There was found to be a higher response (48.5%) from the Dhaka division. The responses were the highest (68.6%) from urban areas. More than half of the participants (52.4%) were students, and 71.28% have passed the Bachelor or equivalent level. The marital status of 67% of respondents is “Single”. Around 12% of the respondents have income less than ten thousand TK.

**Table 1:** Distribution of the demographic characteristics.

	Frequency (n)	Percentage (%)
**Division**		
Dhaka	547	48.49
Chittagong	118	10.46
Rajshahi	125	11.08
Khulna	84	7.45
Barisal	26	2.30
Rangpur	126	11.17
Sylhet	21	1.86
Mymensingh	81	7.18
**Place of residence**		
Urban	774	68.62
Rural	354	31.38
**Religion**		
Muslim	1,007	89.27
Non-muslim	121	10.73
**Age (years)**		
11-20	184	16.31
21-30	854	75.71
31-40	41	3.63
41-50	20	1.77
>50	29	2.57
**Gender**		
Male	622	55.14
Female	506	44.86
**Marital status**		
Single	756	67.02
In a relationship	176	15.60
Married	190	16.84
Widowed/Divorced	6	0.53
**Educational level**		
Higher secondary or below	111	9.84
Bachelor or equivalent	804	71.28
Masters or above	213	18.88
**Employment status**		
Student	592	52.48
Employed	281	24.91
Unemployed	255	22.61
**Income level**		
< 10000 TK	135	11.97
10001-30000 TK	394	34.93
30001-50000 TK	301	26.68
> 50000 TK	298	26.42

### Prevalence of Sleep Disturbance

The prevalence of sleep disturbance by the demographic and personal information of the respondents is presented in **Table 2**. From this web-based survey, it has been observed that 33.24% of the participants claimed to have sleep disturbance during the COVID-19 lockdown. After performing Pearson’s chi-square test of association, it has been observed that place of residence, age, gender, and marital status have a statistically significant association with sleep disturbance. **Table 2** displays that sleep disturbance during this pandemic situation is more common among respondents of urban areas (36.05%) compared to rural areas (27.12%). The highest prevalence of sleep disturbance has been found among respondents aged 31–40 years. Among females, a higher prevalence of sleep disturbance (38.54%) has been observed compared to males (28.94%). The sleep disturbance significantly varies with the marital status of the respondents. Among respondents who were in a relationship, about 40% are suffering from sleep disturbance, whereas the prevalence of sleep disturbance is the lowest (30.56%) among “single” respondents.

**Table 2:** Distribution of sleep disturbance by the respondent’s demographic and personal characteristics.

	Sleep disturbance		*p*-value
	No, n (%)	Yes, n (%)
**Total**	753 (66.76)	375 (33.24)	
**Division**			
Dhaka	351 (64.17)	196 (35.83)	0.247
Chittagong	76 (64.41)	42 (35.59)
Rajshahi	86 (68.80)	39 (31.20)
Khulna	54 (64.29)	30 (35.71)
Barisal	18 (69.23)	8 (30.77)
Rangpur	90 (71.43)	36 (28.57)
Sylhet	18 (85.71)	3 (14.29)
Mymensingh	60 (74.07)	21 (25.93)
**Place of residence**			
Urban	495 (63.95)	279 (36.05)	0.003
Rural	258 (72.88)	96 (27.12)	
**Religion**			
Muslim	672 (66.73)	335 (33.27)	0.963
Non-muslim	81 (66.94)	40 (33.06)	
**Age (years)**			
11-20	132 (71.74)	52 (28.26)	0.034
21-30	568 (66.51)	286 (33.49)
31-40	19 (46.34)	22 (53.66)
41-50	15 (75.00)	5 (25.00)
>50	19 (65.52)	10 (34.48)
**Gender**			
Male	442 (71.06)	180 (28.94)	0.001
Female	311 (61.46)	195 (38.54)	
**Relationship status**			
Single	525 (69.44)	231 (30.56)	0.016
In a relationship	105 (59.66)	71 (40.34)	
Married	121 (63.68)	69 (36.32)	
Widowed/Divorced	2 (33.33)	4 (66.67)	
**Educational level**			
Higher secondary or below	79 (71.17)	32 (28.83)	0.509
Bachelor or equivalent	536 (66.67)	268 (33.33)
Masters or above	138 (64.79)	75 (35.21)
**Employment status**			
Student	402 (67.91)	190 (32.09)	0.644
Employed	182 (64.77)	99 (35.23)
Unemployed	169 (66.27)	86 (33.73)
**Income level**			
< 10000 TK	92 (68.15)	43 (31.85)	0.814
10001-30000 TK	268 (68.02)	126 (31.98)
30001-50000 TK	200 (66.45)	101 (33.55)
> 50000 TK	193 (64.77)	105 (35.23)
**BMI**			
Underweight	66 (63.46)	38 (36.54)	0.792
Normal weight	476 (67.23)	232 (32.77)	
Overweight	170 (65.64)	89 (34.36)	
Obese	38 (70.37)	16 (29.63)	
**Size of family**			
Small	399 (67.40)	193 (32.60)	0.630
Large	354 (66.04)	182 (33.96)	

p-value ≤ 0.001 are treated as 0.001.

**Table 3** explores how sleep quality is disrupted during the COVID-19 lockdown. A higher prevalence of sleep disturbance has been observed among respondents whose family member/relative/friend/neighbor or him/herself has got infected with COVID-19. Among respondents who were working from home or doing online courses (during the lockdown) through the internet, 36.67% have developed sleep disturbance. The prevalence of sleep disturbance varies considerably by internet usage. **Table 3** indicates that the prevalence of sleep disturbance is the highest among respondents whose daily internet use is more than 5 h. Losing the job of any of the family members (including respondents) increases the prevalence of sleep disturbance. Among respondents who thought to be at a high risk of getting infected by COVID-19, 38.46% had a sleep disturbance. It has been observed that about 27% of respondents have claimed to develop anxiety during the lockdown, while 41.8% of them have also claimed to develop sleep disturbance, which is higher than those who do not think themselves as anxious. The sleeping schedule during the COVID-19 lockdown is significantly associated with sleep disturbance. A higher prevalence of sleep disturbance (43.35%) has been observed among respondents who usually sleep more at daytime (6 am–6 pm) than at night (6 pm–6 am) during the lockdown.

**Table 3:** Distribution of sleep disturbance by respondent’s characteristics regarding COVID-19 and new normal life during the lockdown.

	n (%)	Sleep disturbance		*p-*value
		No, n (%)	Yes, n (%)
**Following the social distancing rule**				
No	18 (1.60)	12 (66.67)	6 (33.33)	0.383
Moderately	380 (33.69)	264 (69.47)	116 (30.53)
Strictly	730 (64.72)	477 (65.34)	253 (34.66)
**Infected by COVID-19**				
No	793 (70.30)	548 (69.10)	245 (30.90)	0.010
Yes	335 (29.70)	205 (61.19)	130 (38.81)
**Currently living with family **				
No	84 (7.45)	56 (66.67)	28 (33.33)	0.986
Yes	1,044 (92.55)	697 (66.76)	347 (33.24)
**Working from home/ doing online classes**				
No	629 (55.76)	437 (69.48)	192 (30.52)	0.029
Yes	499 (44.24)	316 (63.33)	183 (36.67)
**Going to the workplace**				
No	618 (82.40)	412 (66.67)	206 (33.33)	0.867
Yes	132 (17.60)	87 (65.91)	45 (34.09)
**Losing job**				
No	909 (80.59)	642 (70.63)	267 (29.37)	0.001
Yes	219 (19.41)	111 (50.68)	108 (49.32)
**Exercise status**				
No	638 (56.56)	414 (64.89)	224 (35.11)	0.129
Yes	490 (43.44)	339 (69.18)	151 (30.82)
**Food consumption dominates** **the new normal life**				
No	738 (65.43)	498 (67.48)	240 (32.52)	0.477
Yes	390 (34.57)	255 (65.38)	135 (34.62)
**Daily internet usage**				
< 3 h	220 (19.50)	155 (70.45)	65 (29.55)	0.004
3-5 h	321 (28.46)	232 (72.27)	89 (27.73)
> 5 h	587 (52.04)	366 (62.35)	221 (37.65)
**Infection risk**				
No	647 (57.36)	457 (70.63)	190 (29.37)	0.001
Yes	481 (42.64)	296 (61.54)	185 (38.46)
**Anxiety**				
No	824 (73.05)	576 (69.90)	248 (30.10)	0.001
Yes	304 (26.95)	177 (58.22)	127 (41.78)
**Sleeping schedule**				
Night	785 (69.59)	558 (71.08)	227 (28.92)	0.001
Day	343 (30.41)	195 (56.85)	148 (43.15)

p-value ≤ 0.001 are treated as 0.001.

### Factors Associated With Sleep Disturbance

Finally, the estimated effects from the logistic regression model for the factors associated with sleep disturbance are presented in **Table 4**. It demonstrates that out of the four significant personal factors of Pearson’s chi-square test, only age and gender remain significant after fitting the logistic regression model. Furthermore, working from home/doing online classes, losing a job, infection risk, anxiety, sleeping schedule remain as significant factors of sleep disturbance. A respondent of age 31 to 40 years has approximately four times higher odds of having a sleep disturbance than a respondent of age 11 to 20 years (AOR: 4.04, 95% CI: 1.77–9.22). Gender has a statistically significant impact on sleep disturbances in favor of males. Compared to male respondents, females are 56% more likely to develop sleep disturbance (AOR: 1.56, CI: 1.19–2.04). The odds of having sleep disturbance is 34% higher among the respondents who are working from home or taking online classes through the internet than those who are not doing so (AOR:1.34, CI: 1.02–1.75). Sleep disturbance is 2.41 times higher among respondents who or anyone from his/her family had lost their job than among the respondents who or anyone from his/her family did not lose their job during lock-down. It has been observed that a respondent who thinks to be at a high risk of getting infected by COVID-19 has 45% higher chance to develop sleep disturbance than a respondent who does not think to be at a high risk of getting infected (AOR: 1.45, 95% CI: 1.11–1.89). Expectedly, anxiety plays a vital role in sleep disturbance. Respondents who are getting anxious during lockdown have 42% greater odds of developing sleep disturbance than respondents who do not think themselves as anxious with an estimated AOR of 1.42 (95% CI: 1.06–1.90). The results suggest that a respondent who sleeps more at daytime (6 am–6 pm) is 86% more likely to develop a sleep disturbance compared to a respondent who sleeps more at night (6 pm–6 am) with an estimated AOR of 1.86 (95% CI: 1.40–2.49).

**Table 4:** Determinant analysis of sleep disturbance: Results from a multivariable logistic regression model.

	AOR	*p*-value	95% CI
**Constant**	0.11	0.001	0.06-0.19
**Place of residence**			
Urban	1.00		
Rural	0.97	0.841	0.71-1.32
**Age (years)**			
11-20	1.00		
21-30	1.41	0.074	0.97-2.05
31-40	4.04	0.001	1.77-9.22
41-50	0.96	0.945	0.29-3.18
>50	1.89	0.215	0.69-5.12
**Gender**			
Male	1.00		
Female	1.56	0.001	1.19-2.04
**Marital status**			
Single	1.00		
In a relationship	1.29	0.164	0.90-1.84
Married	1.08	0.723	0.69-1.70
Widowed/Divorced	2.64	0.281	0.45-15.38
**Infected by COVID-19**			
No	1.00		
Yes	1.11	0.483	0.83-1.49
**Working from home/ doing online classes**			
No	1.00		
Yes	1.34	0.035	1.02-1.75
**Losing job**			
No	1.00		
Yes	2.41	0.001	1.76-3.32
**Daily internet usage**			
< 3 h	1.00		
3-5 h	0.90	0.604	0.59-1.36
> 5 h	1.30	0.183	0.88-1.92
**Infection risk **			
No	1.00		
Yes	1.45	0.006	1.11-1.89
**Anxiety**			
No	1.00		
Yes	1.42	0.019	1.06-1.90
**Sleeping schedule**			
Night	1.00		
Day	1.86	0.001	1.40-2.49

AOR = Adjusted odds ratio and p-value ≤ 0.001 are treated as 0.001.

**Figure 1:**
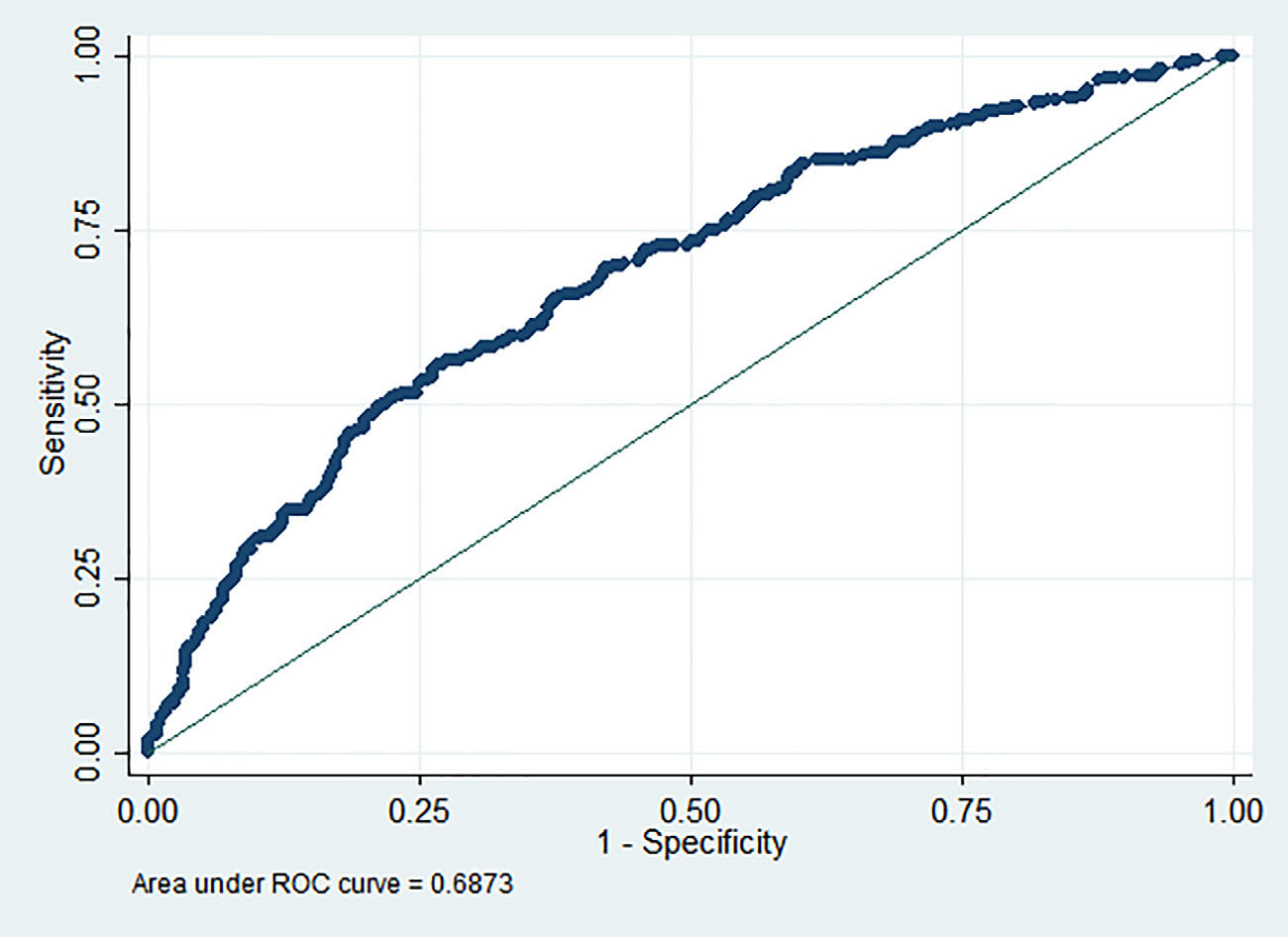
****Performance measure for predicting sleep disturbance using last fitted multivariable logistic regression model.

Lastly, the area under the curve (AUC) of the receiving operating characteristic (ROC) curve has been calculated as a measure of model performance, which explains the model’s performance by evaluating sensitivity *versus* specificity. **Figure 1** displays that there is a 68.7% chance that the final fitted model will be able to distinguish between the positive and negative class of sleep disturbance. Further, statistically significant evidence (p-value <.001) has been found that the model performance measure AUC of the final fitted model is greater than 0.5.

## Discussion

According to Worldometers, a total of 533,441 COVID-19 cases were identified, and the death rate was 16.61% over the cumulative number of closed cases up to 26^th^ March when the first shutdown starts in Bangladesh (5, 54). As of the end of March, infections remained low, but a steep rise had been observed in April 2020 (55). New cases in Bangladesh grew by 1,155% in the week ending on 11^th^ April, which is the highest in Asia, ahead of Indonesia, with 186% (7, 56). This web-based survey shows a high prevalence (33.24%) of sleep disturbance and anxiety (26.95%) during the COVID-19 outbreak. Studies on the Italian population showed a 57.1% prevalence of sleep problems with 32.1% anxiety disorders (57). The Italian study partitioned their region into three geographical segments and revealed sleep disturbance by using official psychological tools. The majority of their respondents (74.3%) were female. They explore the influence of demographic factors and knowledge of people affected by COVID-19 in determining risk for sleep quality. On the other hand, our study assesses how COVID-19 and new normal life during the lockdown disrupts sleep quality. Furthermore, it is impossible to differentiate whether the results of the Italian study are due to the fear of the pandemic or the restrictive measures imposed by the government of Italy. However, the different demographic characteristics of the sample and different aspects of the Italian study made a statistical comparison quite impossible.

Our study indicates that younger participants of age less than 30 years were less likely to develop sleep disturbance compared to older people of age more than 30 years during this lockdown. Around 90% of the respondents aged 31–40 years were living in urban areas where population density is much higher compared to rural areas. As a result, a higher percentage of them (46.34%) thought to be at a high risk of getting infected by COVID-19. This risk of getting infected may disrupt their sleep quality.

Women’s triple burden is depicted across three broad categories of productive, reproductive, and community work (58). Disease outbreak, disaster, or other crises predominantly increase women’s workloads and decrease the ability to balance their time (59–62). Burdens associated with COVID-19 are strenuous, perilous, and likewise gendered. Understanding the extent to which COVID-19 home confinement measures affect women and men differently is fundamental for understanding the broader impact of this disease. During the lockdown, families are at home more which has intensified women’s existing triple burden (63) and fears of violent domestic abusers (64). A recent study has explored women’s triple burden during COVID-19 in three Asian countries (65). Home confinement may have increased household (including caretaking of elderly family members) responsibilities, which may disproportionately affect women. In addition to other jobs, this potential increase in responsibilities during the pandemic may have exacerbated adverse mental health for women in particular. Several studies have reported a higher prevalence of sleep disturbance among women compared to males (19, 57, 66, 67). A recent study in the context of COVID-19 conducted on Bangladeshi people also found a higher prevalence of general anxiety disorder among females (68). Similar to other studies, it has been observed in our study that the odds of getting anxious during the lockdown is about 15% higher among female participants than among males. This high prevalence of anxiety and a greater burden in the household increase the stress of females, which may result in a higher prevalence of sleep disturbance.

It has been observed that sleep disturbance is more common among the participants who are working from home or taking online classes through the internet. One possible explanation behind observing a higher prevalence is that the majority of them (56.31%) are using the internet for more than 5 h a day. Previous studies have identified excessive usage of the internet as a potential source of sleep disorders (69–76). Besides this, around 48% of the respondents who were working from home or taking online classes through the internet were female, which also provides the rationale for observing a higher prevalence among them.

Readymade garments and other export-oriented sectors of Bangladesh had faced a huge shock due to orders cancellation of worth $3.0 billion (77). More than a million have already been fired or furloughed as global fashion companies have canceled or suspended their orders in Bangladesh due to the corona virus crisis (78). The tourism industry of Bangladesh will face a loss of about Tk 40 billion (USD 470 million) this year, whereas this sector directly provided a livelihood for 1.1 million people (79). Around 32 lakh transport workers had become temporarily unemployed (80). It seems that this pandemic now turns into a severe economic crisis as a huge percentage of people have already lost their jobs. Among the participants, 20% stated that their family members or they themselves lost jobs during this lockdown. The finding of this study implies that there is a greater prevalence of sleep disturbance among participants who or anyone from their family members lost jobs during this pandemic as losing jobs create massive insecurity of meeting livelihoods.

The results of this survey have displayed that 42.7% of the participants think that they are at high risk of getting infected. The results of multivariable logistic regression have suggested that the odds of developing sleep disturbance is higher among participants who thought to be at high risk of getting infected. Due to the lockdown, people have been confined to their homes, which results in a severe reduction of their daily social interaction. This lack of social interaction and home confinement may contribute to developing anxiety. Expectedly, it has been observed from the study results that the presence of anxiousness triggers sleep disturbance. In several studies, anxiety had also been found as a potential risk factor of sleep disturbance (81–87). In this study, no statistically significant association (Pearson’s chi-square value = 5.33, *p*-value = 0.255) has been found between respondent’s anxiety and age.

Before the COVID-19 lockdown, the sleeping schedule was more dependent on a person’s working shift. Several studies have found that sleep disorders are more common in night-shift workers compared with day workers (88–90). During lockdown, people have been confined to their homes, which moderately shifts the working hours and work responsibilities. Participants of this study were mainly students (52.48%) and in general, they can have erratic sleep schedules as they have no defined hours to do their work. The results of this study have suggested that sleeping schedule has a statistically significant impact on developing sleep disturbance in favor of participants who slept more at night (6 pm–6 am) than those who slept more at daytime (6 am–6 pm).

The findings of this study suggest that the factors highlighted above can be the potential risk factors of developing sleep disturbance, as previously reported for the Chinese population and accordingly with other studies on epidemic and quarantine conditions (91–94).

### Limitations and Strengths of the Study

The findings of this study should be interpreted in the context of the study’s design and limitations. The study used a web-based non-probabilistic convenience sampling method as it was impossible to take personal interviews due to COVID-19 confinement restrictions. It is always very much difficult to obtain a nationally representative sample through a web-based survey. Expectedly, we have observed more responses from urban areas and comparatively younger groups of Bangladeshi population (mainly students) as they are more active in digital platforms than the people of rural areas and elderly people of Bangladesh. Approximately half of the responses have been obtained from the Dhaka division, which results in failing to capture the regional psychological behavior of the respondents. Since the study is a cross-sectional one, it can only point toward associations. A more robust design such as cohort or case–control is recommended to corroborate causation and generalization. The results of this study could have been more robust if we could have included the information regarding the previous history of sleep disturbance (insomnia), depression, and other mental health conditions (bipolar disorder), the previous history of anxiety disorder, chronic diseases/chronic pain conditions, consumption of medications associated with insomnia.

The survey was basic, with clear categories outlined and no need for official psychological tools, making it easy for respondents to quickly go through the survey. The survey reached people through a variety of digital platforms, making it more accessible and meeting people where they already are (aside from the fact that this was not available in person). Notwithstanding all the limitations, this study explores a relevant topic, mental health and associated conditions like sleeping disorders, which have been identified as one of the secondary effects of the pandemic. To the best of our knowledge, this will be the first study in Bangladesh that assesses the association between sleep disturbance and variables associated with the lockdown prompted by COVID-19.

## Concluding Remarks

Fortunately, many strong national measures have been taken by the Bangladesh government to avoid further spread of the COVID-19 outbreak. However, the public’s psychological problems during the COVID-19 outbreak are still overlooked. This study attempts to fill this research gap by analyzing the prevalence of sleep disturbance as sleep disturbance is one of the key symptoms of major depression and one of the proven risk factors for suicide (95–97). The findings of this study suggest that sleep disturbance during the lockdown is dependent on both demographic characteristics and the COVID-19 related information of the respondents. In conclusion, this study found that variables associated with the lockdown prompted by COVID-19 such as working from home or doing online classes through the internet, job loss, anxiety, fear of infection with COVID-19, and (day) sleeping schedule were predictive factors for developing sleeping disorders in the Bangladeshi population. We anticipate that this framework will help the policymakers to initiate government programs to diagnose and treat mental health disorders due to the secondary impact of the pandemic. We suggest arranging cognitive–behavioral therapy (CBT) which is the most widely-used therapy for sleep disorders and may be conducted individually in a group of people with similar sleeping problems, or even online (98–100). As well as changing the way one’s thinking regarding sleep, CBT also works to change the habits that can prevent someone from sleeping well. A promotional campaign should be arranged to let people know about the digitally available stress and anxiety mitigation resources of WHO (101, 102). Further, researchers may use this study as a means to keep studying the impact of the pandemic in mental health and sleep quality and prompt further interest and investment from the health sector in mental health programs to tackle the impact of COVID-19.

## Data Availability Statement

The raw data supporting the conclusions of this article will be made available by the authors, without undue reservation.

## Ethics Statement

Ethical review and approval was not required for the study on human participants in accordance with the local legislation and institutional requirements. Written informed consent to participate in this study was provided by the participants’ legal guardian/next of kin.

## Author Contributions

Conceived and designed the experiments: TA, MH. Performed the experiments: MH, TA, AA. Analyzed the data: TA, MR. Contributed reagents/materials/analysis tools: TA, MR. Wrote the paper: MR, TA, AA. All authors contributed to the article and approved the submitted version.

## Conflict of Interest

The authors declare that the research was conducted in the absence of any commercial or financial relationships that could be construed as a potential conflict of interest.
